# Investigating the risk of breast cancer among women exposed to chemicals: a nested case–control study using improved exposure estimates

**DOI:** 10.1007/s00420-019-01479-4

**Published:** 2019-10-24

**Authors:** Cecilia Videnros, Jenny Selander, Pernilla Wiebert, Maria Albin, Nils Plato, Signe Borgquist, Jonas Manjer, Per Gustavsson

**Affiliations:** 1 Unit of Occupational Medicine, Institute of Environmental Medicine, Karolinska Institutet, Solnavägen 4, 113 65 Stockholm, Sweden; 2 Centre for Occupational and Environmental Medicine, Stockholm County Council, Stockholm, Sweden; 3 Department of Clinical Sciences Malmö, Lund University, Malmö, Sweden; 4 Department of Surgery, Skåne University Hospital, Malmö, Sweden

## Abstract

**Purpose:**

The aim of this study was to examine if exposures to chemicals at the workplace were associated with an increased risk of postmenopausal breast cancer, using improved exposure estimates.

**Methods:**

The design is a case–control study, nested within a cohort of women from the Malmö Diet and Cancer Study. The study comprised 2400 women, 731 cases and 1669 matched controls, born 1923–1950 and living in Malmö, Sweden between 1991 and 1996. An occupational hygienist reclassified the probability for exposure given by a job-exposure matrix, using individual data on work tasks. First-time diagnoses of invasive breast cancer were identified through the Swedish Cancer Registry.

**Results:**

Women exposed to chemicals in their occupational environment had a statistically significantly increased risk (OR 1.59, 95% CI 1.11–2.29) of breast cancer, and the risk correlated positively with duration of exposure but not with exposure intensity. Women exposed to chlorinated hydrocarbon solvents for more than 10 years had a significant higher risk of breast cancer (OR 3.06, 95% CI 1.18–7.96) as well as women exposed to oil mist for more than 10 years (OR 3.08, 95% CI 1.12–8.49).

**Conclusions:**

This study gives some support to the hypothesis that exposure to organic solvents as well as oil mist is associated with increased risk of breast cancer.

## Introduction

With 1 million new cases diagnosed in the world each year, breast cancer is the most common cancer among women globally (McPherson et al. 2000). Breast cancer accounts for 12% of all cancers in the world and 25% of all cancers among women (Ferlay et al. 2015).

Established risk factors for breast cancer include hormonal factors related to reproduction, such as early menarche, late menopause, late first-time pregnancy, nulliparity, oral contraceptive use, and hormone replacement therapy (HRT) (Schottenfeld and Fraumeni 2006). Other risk factors are alcohol consumption (Longnecker 1994), family history of breast cancer (Morrow and Gradishar 2002), and high body mass index (BMI) (Schottenfeld and Fraumeni 2006).

An association between chemical exposure and breast cancer has been suggested, acting through three different mechanisms; (i) genotoxic action, (ii) alteration of mammary gland development or hormone responsiveness, and (iii) hormonal tumour promotion.(Rodgers et al. 2018) Organic solvents have been hypothesised to increase the risk of breast cancer in occupations like dry cleaners, painters, and laboratory technicians (Goldberg and Labreche 1996; Hansen 1999; Peplonska et al. 2010; Gustavsson et al. 2017; Ekenga et al. 2014; Labreche and Goldberg 1997). Lifetime cumulative exposure to organic solvents among men has been linked to increased risk of male breast cancer (Laouali et al. 2018). Organic solvents are absorbed through the mucous membranes in the respiratory system or through the skin and are then spread throughout the body via the circulatory system (Labreche and Goldberg 1997). The mechanism for organic solvents to cause breast cancer is not fully understood, but is thought to be directly by acting as a genotoxic agent or indirectly through their metabolites (Rodgers et al. 2018; Dumitrescu and Shields 2005).

Other chemical exposures that have been of interest are endocrine-disrupting chemicals (EDCs). The EDCs can alter the breast development or promote tumour growth (Rodgers et al. 2018). Examples of EDC chemicals are ethylene oxide, polycyclic aromatic hydrocarbons (PAH), and some pesticides which occur in occupations like painters, welders, and horticultural workers (Fenga 2016).

Our previous study, using the same cohort, estimated exposure using a job-exposure matrix and found increased risk of postmenopausal breast cancer for women exposed to chemicals (Videnros et al. 2019). Organic solvents seemed to be associated with an increased risk of postmenopausal breast cancer, although with a non-significant hazard ratio. A potential drawback of that study was the misclassification of exposure introduced using a job-exposure matrix. The aim of this study was to examine if exposure to chemicals at the workplace was associated with an increased risk of postmenopausal breast cancer, using improved exposure estimates based on a case-by-case blinded evaluation of the individual probability of exposure performed by an occupational hygienist.

## Materials and methods

The present case–control study is nested within the cohort study Malmö Diet and Cancer Study (MDCS) which is described in detail elsewhere (Manjer et al. 2001; Kullberg et al. 2017). Briefly, MDCS includes 28,098 participants (of which 17,035 were women) born 1923–1950 who lived in Malmö, Sweden during the recruitment years 1991–1996. The original aim of the research project was to investigate the relationship between dietary habits and the risk of cancer.

The present study intended to include all 1088 cases from the MDCS cohort. However, 239 cases were excluded due to missing questionnaires that were needed for the individual exposure assessment. The questionnaires for these 239 cases were accidently lost before the detailed work information could be extracted. However, sensitivity analysis showed no significant difference between the women with lost questionnaires and the rest of the sample, and thus, no bias were likely introduced by excluding these cases from the study. This resulted in a population of 2547 participants, 849 breast cancer cases, and 1698 controls. Each case was matched with two controls on age and the selection of controls was density based. Exclusion criteria for this study were women with no self-reported work history (*n* = 42), diagnosis of breast cancer before baseline (*n* = 50), and premenopausal status until end of follow-up (*n* = 55). Since risk factors for pre- and postmenopausal breast cancer differ in some aspects, all premenopausal breast cancer cases were excluded (Schottenfeld and Fraumeni 2006). E.g., high BMI is a risk factor for postmenopausal breast cancer, while it is a protective factor for premenopausal breast cancer. Also, the few cases of premenopausal women in the cohort made the group too small to analyse. Menopausal status was defined by a combination of questionnaire data and medical records. A women was classified postmenopausal if she: (i) had undergone bilateral oophorectomy or (ii) was 55 years or older and had undergone hysterectomy or (iii) the above criteria was missing and she confirmed that her menstruation had stopped 2 years prior to baseline or (iv) the above criteria was missing and she was 55 years or older. A total of 2400 women were left in the nested case–control population, 731 cases and 1669 controls.

At recruitment, each participant filled out an extensive questionnaire with questions on lifestyle, reproductive factors and working history. Each woman reported her three latest occupations, the time period for these and specific tasks in each occupation. The women had each occupation for an average of 18 years, leading to an almost complete collection of their working life. Health care personnel measured weight and height for each woman. The validated questionnaire AUDIT was used to estimate the alcohol consumptions (Piccinelli et al. 1997).

Exposure to chemicals was assessed through a combination of the Scandinavian job-exposure matrices NOCCA and FINJEM and an occupational hygienists estimation of every woman’s probability of exposure according to the specific work task specified in the baseline questionnaire (Kauppinen et al. 1998, 2009). The JEM specifies an exposure intensity level (*I*) and a prevalence of exposure (*P*) for 300 occupations. The occupational hygienist reviewed and adjusted the prevalence estimate (*P*) for each woman’s exposure based on the description in the questionnaire. If a woman was considered as unexposed the prevalence was set to 0%, and if exposure was likely or certain it was set to 100%. The original prevalence was retained if the case-by-case evaluation did not indicate otherwise. The prevalence set by the occupational hygienist substantially increased the accuracy of the exposure estimates. Exposure estimates used in this paper were “ever exposed” (*p* > 0),” mean intensity” (*I* × *P* × years worked in the exposed occupation/total working years, measured in mg/m^3^ or ppm) and duration of exposure (years worked in the exposed occupation). Chemicals of interest for our study that were available in NOCCA was; 1,1,1-trichloroethane, benzene, benzo(a)pyrene, bitumen fumes, diesel exhaust, gasoline exhaust, methylene chloride, perchloroethylene, toluene, and trichloroethylene. Chemicals used from FINJEM were aliphatic and alicyclic hydrocarbon solvents, aromatic hydrocarbon solvents, chlorinated hydrocarbon solvents, other organic solvents (including alcohols, ketones, esters, glycol ethers, etc.), fungicides, herbicides, insecticides, polycyclic aromatic hydrocarbons (PAH), gasoline exhaust, and oil mist. Ever exposed to chemicals in our study was defined as having worked in an occupation where at least 5% of the employees were, according to the matrices, exposed to any of these chemicals. Exposure from participants’ three latest occupations was added and calculated up until the women were enrolled in the study. The age range of inclusion was 45–74 years of age with a mean age of 58 years, resulting in an almost complete working history for each woman.

First-time invasive breast cancer diagnoses between 1991 and 2013 were identified through the Swedish Cancer Registry, with a coverage of 99% of all Swedish breast cancer cases (Barlow et al. 2009). Breast cancer cases were identified as ICD7 code = 170 (International Classification of Diseases, 7th Revision). Death and migration status were collected from Swedish National Tax Board.

### Statistical analyses

Confounders to include in the analyses were selected based on a priori knowledge and statistical analysis described in detail in a previous paper (Kullberg et al. 2017). Confounding risk factors were age (45–49, 50–54, 55–59, 60–64, 65–69, 70–74), age at first-term pregnancy (< 20, 20–24, 25–29, 30–34, 35+), parity (0, 1, 2, 3, ≥ 4), months of breastfeeding each child (0, 1–5, 6–12, ≥ 13), hormone replacement therapy (HRT) (no treatment, estrogen, progesterone, combined treatment), height (< 160, 160–169, ≥ 170 cm), physical activity (quartiles), alcohol consumption (0, 1–14, 15–30, > 30 g/day), and BMI (< 18.5, 18.5–24.9, 25.0–29.9, ≥ 30). Variables considered but rejected from the model were family history of any cancer (due to too general variable), smoking (due to inconsistency in the literature and no statistical association), and education (due to collinearity with occupation) (Kullberg et al. 2017).

BMI was calculated as kg/m^2^ and categorised according to the WHO standard (World Health Organization 1995). Physical activity outside work was measured with questions estimating the time of physical activity performed and then multiplied with an intensity factor specific for each activity. A number of months of breastfeeding were reported and women’s mean number of months were imputed if having missing on one child’s breastfeeding data.

Chi-square test was used to compare the distribution of risk factors for breast cancer among the cases and controls. Both conditional and unconditional logistic regression was calculated to estimate the odds ratio (OR) for breast cancer in women exposed to chemicals/never exposed women and in duration analysis of 1–10 years and > 10 years. Since there was no significant difference between the results, we used unconditional logistic regression for improved power. Crude estimates were adjusted for age only and the adjusted model included age, parity, age at first-term pregnancy, months of breastfeeding/child, HRT, physical activity, alcohol consumption, height, and BMI. Mean intensity was divided dichotomously at the median and analysed using logistic regression. Trend tests in Tables 2 and 3 were calculated using logistic regression, creating a variable assigning the value 0 to the unexposed group, 1 to the low/short exposed group, and 2 to the high/long exposed group with the unexposed group as a reference. Pearson correlation analysis was used to investigate the correlations between chemical agents and main chemical groups, since women who are exposed to one chemical agent probably are exposed to others. Statistical analyses were performed using STATA (version 13.0) with an α-level for significance tests at 0.05 (Stata 2013).

The MDCS study was approved by Lund ethical review board (LU 51-90) and the present study was approved by the Stockholm ethical review board (Dnr 2014/233-31/4).

## Results

The nested case–control study comprised 2400 women, 731 cases and 1669 controls. Table 1 shows the distribution of risk factors for breast cancer in cases and controls. The cases were significantly older, used HRT to a higher extent, drank more alcohol and breastfed for a longer time compared to the controls.

**Table 1:** Distribution of potential and established risk factors for breast cancer among cases and controls

	Cases *n* = 731	(%)	Controls *n* = 1 669	(%)	Chi-square test^a^
Age^b^ (years)					*p *= *0.002*
45–49	95	13.0	333	20.0	
50–54	110	15.1	258	15.5	
55–59	208	28.5	411	24.6	
60–64	170	23.3	365	21.9	
65–69	86	11.8	181	10.8	
70–74	62	8.5	121	7.3	
Parity					*p *= *0.153*
0	101	14.2	211	12.8	
1	139	19.5	360	21.9	
2	311	43.7	665	40.4	
3	125	17.6	292	17.7	
≥ 4	36	5.1	118	7.2	
Age at first-term pregnancy					*p *= *0.690*
< 20	63	8.9	170	10.3	
20–24	248	34.8	557	33.9	
25–29	200	28.1	479	29.1	
30–34	75	10.5	181	11.0	
35+	25	3.5	46	2.8	
No children	101	14.2	211	12.8	
Months of breastfeeding/child					*p *= *0.002*
0	24	3.5	83	5.2	
1–5	343	50.3	890	55.8	
6–12	205	30.1	399	25.0	
≥ 13	9	1.3	11	0.7	
No children	101	14.8	211	13.2	
Hormone replacement therapy					*p *< *0.001*
No treatment	508	70.5	1325	81.1	
Estrogen	52	7.2	119	7.3	
Progesterone	4	0.6	8	0.5	
Estrogen + Progesterone	157	21.8	181	11.1	
Physical activity (percentile)					*p *= *0.588*
0–25	182	25.0	391	23.5	
25–50	201	27.6	434	26.1	
50–75	176	24.2	419	25.2	
75–100	169	23.2	419	25.2	
Alcohol (g/day)					
0	35	4.9	121	7.4	*p *= *0.002*
1–14	559	77.4	1259	76.6	
15–30	101	14.0	236	14.4	
> 30	27	3.7	27	1.6	
Height (cm)					*p *= *0.289*
< 160	157	21.5	407	24.4	
160–169	460	62.9	1008	60.5	
≥ 170	114	15.6	250	15.0	
BMI					*p *= *0.095*
< 18.5 Underweight	8	1.1	12	0.7	
18.5–24.9 Normal weight	323	44.2	825	49.6	
25–29.9 Overweight	276	37.8	570	34.2	
≥ 30 Obese	124	17.0	258	15.5	

Italic values are statistically significant at p < 0.05

^a^Chi-square test comparing the distribution of potential and established risk factors for breast cancer among the cases and controls

^b^Age at baseline

Table 2 shows that women exposed to any of the included chemicals had a statistically significant increased risk of breast cancer compared to unexposed women (OR 1.59, 95% CI 1.11–2.29). Specifically, being exposed to any of the chemicals for > 10 years was associated with an increased OR of 1.88 (95% CI 1.20–2.96). A trend test showed an increased risk of breast cancer with increased duration of exposure to any of the chemicals (*p* = 0.01).

**Table 2:** Odds ratio for invasive breast cancer by exposure duration; ever exposed, 1–10 years, and > 10 years of exposure

Chemical agents	N total	N cases	Ever exposed Crude^g^		Ever exposed Adjusted^h^		N cases	1–10 years exposure Adjusted^h^		N cases	>10 years exposure Adjusted^h^		Trend test
			OR	95% CI	OR	95% CI		OR	95% CI		OR	95% CI	*p* value
Any of the included chemicals^a^	106	58	1.28	(0.92–1.79)	1.59	(1.11–2.29)	20	1.24	(0.70–2.19)	38	1.88	(1.20–2.96)	*0.01*
Organic solvents^b^	96	32	1.12	(0.72–1.73)	1.37	(0.85–2.21)	12	0.99	(0.48–2.02)	20	1.83	(0.96–3.49)	*0.10*
Aromatic hydrocarbon solvents^c^	44	16	1.12	(0.61–2.05)	1.34	(0.69–2.58)	5	0.74	(0.24–2.33)	11	1.92	(0.83–4.42)	*0.22*
Chlorinated hydrocarbon solvents^d^	28	16	1.25	(0.67–2.32)	1.91	(0.97–3.76)	6	1.17	(0.43–3.19)	10	3.06	(1.18–7.96)	*0.03*
*1,1,1*-*trichloroethane*	34	10	1.06	(0.50-2.24)	1.17	(0.53–2.56)	2	0.60	(0.13–2.89)	8	1.55	(0.61–3.94)	*0.51*
Other organic solvents^e^	40	16	1.45	(0.76–2.76)	1.51	(0.76–3.00)	8	1.42	(0.58–3.47)	8	1.65	(0.57–4.79)	*0.24*
Fumes^f^	35	13	1.43	(0.71–2.86)	1.72	(0.84–3.54)	6	1.80	(0.61–5.33)	7	1.66	(0.64–4.34)	*0.17*
Diesel exhaust	24	9	1.49	(0.64–3.44)	1.83	(0.76–4.40)	4	2.49	(0.59–10.45)	5	1.52	(0.49–4.68)	*0.26*
Oil mist	42	15	1.26	(0.66–2.39)	1.76	(0.89–3.48)	6	1.09	(0.41–2.89)	9	3.08	(1.12–8.49)	*0.04*

Italic values are statistically significant at p < 0.05

^a^Exposed in the work environment to at least one of the following chemicals; aliphatic and alicyclic hydrocarbon solvents, aromatic hydrocarbon solvents, chlorinated hydrocarbon solvents, other organic solvents, fungicides, herbicides, insecticides, polycyclic aromatic hydrocarbons, bitumen fumes, diesel exhaust, gasoline exhaust, oil mist, benzene, trichloroethylene, toluene, perchloroethylene, 1,1,1-trichloroethane, gasoline, benzo(a)pyrene, methylene chloride

^b^Aliphatic and alicyclic hydrocarbon solvents, aromatic hydrocarbon solvents, chlorinated hydrocarbon solvents, other organic solvents

^c^Benzene, touluene

^d^Methylene chloride, perchloroethylene, 1,1,1-trichloroethane, trichloroethylene

^e^Includes alcohols, ketones, esters, glycol ethers, etc.

^f^Polycyclic aromatic hydrocarbons, bitumen fumes, diesel exhaust, gasoline exhaust

^g^Adjusted for age

^h^Adjusted for age, parity, age at first-term pregnancy, months of breastfeeding per child, hormonal replacement therapy, physical activity, alcohol consumption, height, and BMI

In the ever/never analysis in Table 2, all estimates for chemical groups or specific chemicals show an increased risk of breast cancer, although only the category “any of the included chemicals” showed a statistically significantly increased OR. On the other hand, when analysing duration of exposure several statistically significant ORs became apparent in those exposed > 10 years. Women exposed to chlorinated hydrocarbon solvents > 10 years had a significantly increased risk of breast cancer (OR 3.06, 95% CI 1.18–7.96). There was a statistically significant trend of increasing duration of exposure to chlorinated hydrocarbon solvents and risk of breast cancer (*p* = 0.03). Also, women exposed to oil mist > 10 years had a statistically increased risk of breast cancer (OR 3.08, 95% CI 1.12–8.49) with a statistical significant trend of increasing duration of exposure to oil mist and risk of breast cancer (*p* = 0.04).

When investigating the risk by mean intensity for each chemical in Table 3, there was no clear overall trend of increasing risk of breast cancer with increasing mean intensity. Women exposed to a high mean intensity of chlorinated hydrocarbon solvents had a non-significant OR of 1.61 (95% CI 0.57–4.58) and there was no indication of a trend. Women exposed to a high intensity of oil mist had an OR of 2.70 (95% CI 1.09–6.68) compared to unexposed women, and there was a statistically significant trend of increasing risk of breast cancer with increased intensity of exposure to oil mist.

**Table 3:** Odds ratio for invasive breast cancer by mean intensity exposure to chemicals

Chemical agents	Mean intensity^f^ to exposure in class	n cases	Crude^g^		Adjusted^h^		Trend test
			OR	95% CI	OR	95% CI	*p* value
Organic solvents^a^ (ppm)							
Unexposed	0	699	1		1		*0.35*
> 0–1.24	0.67	11	1.15	(0.55–2.39)	2.07	(0.90–4.77)	
2.00–109.10	11.75	21	1.10	(0.65–1.88)	1.14	(0.64–2.04)	
Aromatic hydrocarbon solvents^b^ (ppm)							
Unexposed	0	715	1		1		*0.68*
> 0–0.69	0.31	8	1.49	(0.60–3.67)	2.28	(0.83–6.28)	
2.00–36.79	12.32	8	0.89	(0.39–2.04)	0.91	(0.37–2.23)	
Chlorinated hydrocarbon solvents^c^ (ppm)							
Unexposed	0	715	1		1		*0.10*
> 0–0.50	0.35	10	1.60	(0.70–3.69)	2.17	(0.89–5.27)	
0.56–6.60	1.85	6	0.91	(0.35–2.37)	1.61	(0.57–4.58)	
1,1,1-trichloroethane (ppm)							
Unexposed	0	721	1		1		*0.76*
> 0–0.41	0.32	5	1.20	(0.42–3.49)	1.23	(0.42–3.63)	
0.47–1.34	0.83	5	0.94	(0.33–2.69)	1.10	(0.36–3.39)	
Other organic solvents^d^ (ppm)							
Unexposed	0	715	1		1		*0.18*
> 0−2.00	2.00	7	1.23	(0.54–2.82)	1.22	(0.48–3.06)	
2.34–52.50	10.39	9	1.87	(0.67–5.20)	2.01	(0.72–5.64)	
Fumes^e^ (mg/m^3^)							
Unexposed	0	718	1		1		*0.12*
> 0–0.05	0.03	6	1.33	(0.49–3.65)	1.41	(0.50–4.00)	
0.05–10.03	1.80	7	1.52	(0.58–3.96)	2.07	(0.77–5.59)	
Diesel exhaust (mg/m^3^)							
Unexposed	0	722	1		1		*0.25*
> 0–0.03	0.02	5	1.75	(0.55–5.60)	2.16	(0.64–7.34)	
0.03–0.05	0.04	4	1.26	(0.37–4.22)	1.53	(0.44–5.43)	
Oil mist (mg/m^3^)							
Unexposed	0	716	1		1		*0.04*
> 0–0.09	0.06	5	0.74	(0.26–2.04)	1.03	(0.35–2.99)	
0.09–1.80	0.46	10	1.94	(0.83–4.52)	2.70	(1.09–6.68)	

Italic values are statistically significant at p < 0.05

^a^Aliphatic and alicyclic hydrocarbon solvents, aromatic hydrocarbon solvents, chlorinated hydrocarbon solvents, other organic solvents

^b^Benzene, toluene

^c^Methylene chloride, perchloroethylene, 1,1,1-trichloroethane, trichloroethylene

^d^Includes alcohols, ketones, esters, glycol ethers, etc.

^e^Polycyclic aromatic hydrocarbons, bitumen fumes, diesel exhaust, gasoline exhaust

^f^Mean intensity calculated as: intensity level stated in NOCCA/FINJEM × proportion exposed × years worked in the exposed occupation/total working years

^g^Adjusted for age

^h^Adjusted for age, parity, age at first child, months of breastfeeding per child, hormonal replacement therapy, physical activity, alcohol consumption, height, and BMI

The correlation was low between the main chemical groups organic solvents, fumes, pesticides, and oil mist (*r* = 0.01–0.38). However, when analysing individual chemical agents, the correlation was high for diesel and gasoline engine exhaust (*r* = 0.61), methylene chloride and 1,1,1-trichloroethane (*r* = 0.87), and fungicides and insecticides (*r* = 0.87).

## Discussion

This study showed that women exposed to chemicals in their work environment had an increased risk of breast cancer compared to unexposed women. Specifically, women exposed to chemicals for more than 10 years had an almost doubled risk of breast cancer compared to unexposed women. There was a statistically significant trend of duration of exposure to chemicals with the risk of breast cancer. Furthermore, women exposed longer than 10 years to chlorinated hydrocarbon solvents or oil mist had a three times increased risk of developing breast cancer.

The main analysis in Table 2 shows a statistically significant increased risk of breast cancer for women exposed to chemicals in their work environment (OR 1.59, 95% CI 1.11–2.29). There was a significant trend (*p* = 0.01) of increasing time of exposure with increasing risk of breast cancer and women exposed to chemicals more than 10 years had an increased odds ratio of 1.88 (95% CI 1.20–2.96). These findings are in line with our previous finding investigating the entire cohort using a job-exposure matrix for exposure assessment (Videnros et al. 2019).

When investigating what particular chemicals that contribute to an increased risk of breast cancer, chlorinated hydrocarbon solvents showed to be statistically significant. Women exposed to chlorinated hydrocarbon solvents for more than 10 years had an odds ratio of 3.06 (95% CI 1.18–7.96) of developing breast cancer compared to unexposed women. These findings support previous studies on exposure to organic solvents and increased risk of breast cancer (Goldberg and Labreche 1996; Hansen 1999; Peplonska et al. 2010; Gustavsson et al. 2017; Ekenga et al. 2014; Labreche and Goldberg 1997). We found a slightly increased risk of breast cancer in association with exposure to chlorinated hydrocarbon solvents in our previous study, although not statistically significant (Videnros et al. 2019). Our analysis of breast cancer risk and mean intensity showed a non-significant increased risk both in the low-intensity group (OR 2.17, 95% CI 0.89–5.27) and in the high-intensity group (OR 1.61, 95% CI 0.57–4.58). Duration of exposure to organic solvents seem to be a more important determinant for breast cancer than mean intensity of exposure in this study.

Exposure to oil mist was associated with increased risk of breast cancer especially in women exposed for more than 10 years (OR 3.08, 95% CI 1.12–8.49). Exposure to oil mist among females in Sweden occurs mainly in textile work and could be an exposure from spinners’ oil in spinning machines or dyeing processes. Women exposed to a high intensity of oil mist with a mean average intensity of 0.46 mg/m^3^ had an OR of 2.70 (95% CI 1.09–6.68) compared to unexposed women with a statistically significant trend (*p* = 0.04). This is notable, since the occupational exposure limit for oil mist today in Sweden is 1.00 mg/m^3^ (Swedish Work Environment Authority 2018). Sweden has a relatively low limit compared to, e.g., USA with a permissible exposure limit at 5.0 mg/m^3^ (NIOSH pocket guide to chemical hazards 2007). If further studies see similar findings, it would indicate that the occupational exposure limit for oil mist in the work environment does not protect women from breast cancer. Both the high intensity and the long duration of exposure seem to be harmful. However, these results should be interpreted with caution and need to be further investigated. The confidence intervals were quite wide with relatively few exposed cases, which could indicate a chance finding. Furthermore, previous literatures on breast cancer and oil mist are sparse, and the studies that exist do not show an association between oil mist and breast cancer (Weiderpass et al. 1999; Hayes et al. 1993).

This study is based on the same cohort as our previous study of occupational chemical exposures and the risk of postmenopausal breast cancer (Videnros et al. 2019). Our intention was to create an even better study using improved exposure estimates from an occupational hygienist. This study used a case–control design instead of a cohort design to make it feasible for the occupational hygienist to classify the exposure for each woman individually. When comparing the results from these two studies, it is clear that the OR for women ever exposed to chemicals and also specific chemical agents have increased after the occupational hygienist made new exposure assessments. The OR for women ever exposed to any of the chemicals we included have increased from 1.26 (95% CI 1.02–1.54) (Videnros et al. 2019) to an OR of 1.59 (95% CI 1.11–2.29) in this study. This indicates that the JEM used in our previous study probably introduced a misclassification of exposure leading to an attenuation of the results towards an OR of 1.00. However, the JEM still showed results in line with the results using individual exposure estimates, indicating that a JEM is a good enough tool to use when individual estimates are not available.

This study has several strengths and weaknesses that are discussed here in further detail. This study is unique in its way of containing extensive individual information on hormonal and reproductive factors allowing for a good confounding control. Since most of the confounders were strongly negative, failing to adjust for these would underestimate the real risk or failing to see any association at all which is the results many previous studies have found. Hormonal replacement treatment and alcohol consumption were the strongest negative confounders. The outcome measurement, breast cancer diagnosis, is very accurate, since all cancer cases in Sweden are obligated to be reported to the Swedish Cancer registry, resulting in a close to 100% coverage. Another strength is the updated and improved exposure assessment. An occupational hygienist has carefully evaluated all exposed women’s work tasks and specifications about her work to estimate the exposure to different chemicals. Allowing for individual exposure assessments instead of exposure assessment on group level helps to reduce the risk of misclassification of exposure and is, therefore, a superior method. However, even if using best available data and resources for exposure assessment, there is a slight possibility for misclassification of exposure. The exposure was estimated from work description rather than actual measurements on site which contribute to misclassification. Nevertheless, the misclassification is probably non-differential, since job description was specified before breast cancer diagnosis and the occupational hygienist was blinded, leading only to a possible attenuation of the results towards an OR of 1.00. Another drawback is the work history that was only available up until the day of enrolment to the study. Though, the participants were quite old (mean age 58) at recruitment and many were close to retirement, leaving an almost complete occupational exposure history for most participants. Another weakness in this study is the power. Only two controls per case for this study were feasible to examine for the occupational hygienist, where more controls would have been optimal to get the best power (Woodward 2005). The exposures which we study are quite rare which makes the power low, especially for sub-analyses. Also, the 239 lost questionnaires lead to a decrease in study population and, therefore, decrease in power. However, when investigating what questionnaires were lost, no overrepresentation in any characteristics where found and thus should not lead to any bias in the study.

In conclusion, this study gives some support to the hypothesis that exposure to organic solvents as well as oil mist are associated with increased risk of breast cancer. The exposure assessment based on a case-by-case review of exposure gave slightly higher risk estimates for some exposures than a previous analysis based on a job-exposure matrix.
